# Can an *IL13* -1112 C/T (rs1800925) polymorphism predict responsiveness to neoadjuvant chemoradiotherapy and survival of Chinese Han patients with locally advanced rectal cancer?

**DOI:** 10.18632/oncotarget.9178

**Published:** 2016-05-04

**Authors:** Lin Xiao, Xin Yu, Rong Zhang, Hui Chang, Shaoyan Xi, Weiwei Xiao, Zhifan Zeng, Huizhong Zhang, Ruihua Xu, Yuanhong Gao

**Affiliations:** ^1^ Department of Radiation Oncology, Sun Yat-sen University Cancer Center, Guangzhou, China; ^2^ State Key Laboratory of Oncology in South China, Collaborative Innovation Center of Cancer Medicine, Guangzhou, China; ^3^ Department of Oncology, Section II, Jiangmen Central Hospital, Affiliated Jiangmen Hospital of Sun Yat-sen University, Jiangmen, China; ^4^ Department of Endoscopy and Laser, Sun Yat-sen University Cancer Center, Guangzhou, China; ^5^ Department of Pathology, Sun Yat-sen University Cancer Center, Guangzhou, China; ^6^ Department of Medical Oncology, Sun Yat-sen University Cancer Center, Guangzhou, China

**Keywords:** interleukin-13, single-nucleotide polymorphism, locally advanced rectal cancer, neoadjuvant chemoradiotherapy, prognosis

## Abstract

We sought to determine whether a polymorphism in the Interleukin 13 gene (*IL13*), 1112 C/T (rs1800925) predicts responsiveness to neoadjuvant chemoradiotherapy (neoCRT) and prognosis in Chinese Han patients with locally advanced rectal cancer (LARC). Pre-treatment biopsies of primary rectal lesion and surgical specimens were collected from 58 patients with LARC, who were treated with neoCRT and surgery. Tumor DNA was extracted from these biopsies and sequenced to analyze the rs1800925 polymorphism. The tumor response to neoCRT was categorized using a tumor regression grade (TRG, 0-2 were poor responders; 3-4 were good responders). Analyses of progression free survival (PFS) and overall survival (OS) were carried out using the Kaplan-Meier method. Of the forty-six patients for whom tumor DNA was successfully sequenced, 23 were good responders to neoCRT (11 patients with a pathological complete response, i.e. pCR) and the other 23 were poor responders. Good and poor responders were equally likely to have a C/C genotype at rs1800925 (73.9%) as a T/T or C/T genotype (26.1%). There were no differences between the C/C and T/T+C/T genotypes with respect to the ypT0-2 ratio (38.2% vs. 41.7%, *P* = 1.0), ypN0 nodal status (67.6% vs. 50.0%, *P*= 0.314), 6-year PFS (67.6% vs. 50%, *P*=0.274), or 6-year OS (76.5% vs. 66.7%, *P*=0.441). Thus, the IL13-1112 C/T (rs1800925) polymorphism does not predict responsiveness to neoCRT or prognosis of Chinese Han patients with LARC.

## INTRODUCTION

Rectal cancer is one of the ten most common cancers and is a leading causes of cancer death in China [1]. Neoadjuvant chemoradiotherapy (neoCRT) followed by total mesorectal excision (TME) is currently the standard treatment for locally advanced rectal cancer (LARC, i.e T3~T4/N+), resulting in improved local control, R0 resection, and pathological tumor downstaging [2]. However, neoCRT is not equally beneficial for all patients, as 12% to 24% of patients have a minimal response to neoCRT, while 10% to 34.7% of patients have complete loss of residual cancer cells in surgical specimens, i.e. pathological complete response (pCR) [3–6]. Patients with pCR have a better prognosis than those with non-pCR [2, 5]. Thus, identification of predictive markers of cancer response to neoCRT is of clinical importance for personalized therapy. For those with a priori cancer resistant to chemoradiotherapy, more intensive preoperative treatment regimens may be better than conventional neoCRT [7]. In contrast, for patients achieving a clinical complete response (cCR) after neoCRT, radical surgery may be potentially spared or deferred with the help of strict observation [8–10].

How to accurately predict the responsiveness to neoCRT still remains a challenge [11]. A series of clinical parameters such as radiation dose, concomitant chemotherapy protocols [11, 12], pre-treatment serum carcinoembryonic antigen (CEA) level [13], interval between completion of neoCRT and surgery [14, 15], and complication of Diabetes Mellitus [16] has been used to predict tumor responsiveness to neoCRT. Many studies have been carried out to seek predictive biological biomarkers of tumor response to neoCRT, and have mostly focused on a series of proteins involved in apoptosis (Smac [17] and Bax [18]), cell cycle arrest (p21), and distant metastasis (MMP-9 [19]). However, none of these targets have been validated and incorporated into clinical practice. One promising approach to finding key molecular markers is to study single nucleotide polymorphisms (SNPs) or mutations of cancer related genes [7, 11, 20–21].

Cytokines are one of the most common components of tumor inflammatory microenvironment, especially for colorectal cancers [22]. *IL13*, one of many immune inhibitory cytokines generated by Th2 lymphocytes, is involved in tumor development by inhibiting immune surveillance functions of natural killer cells, CD4 (+) Th1 cells and CD8 (+) cytotoxic T lymphocytes [23, 24]. There are several SNPs in the *IL13* gene, such as a functional polymorphism, rs1800925, located in the promoter, rs2066960 in intron 1, rs1295686 in intron 3, rs20541 in exon 4, and rs1295685 in exon 4 [25]. Recently, there has been more interest in rs1800925 (1112 C/T) since it was found that a C/T genotype at this locus was associated with a higher risk of colon cancer occurrence in the Polish population [26]. C/T and T/T genotype were also associated with a higher risk of colorectal cancer and inflammatory bowel diseases, such as ulcerative colitis and Crohn's disease [27]. This SNP was also associated with tumor response to neoCRT in Caucasian patients with LARC, with patients harboring the T allele having a poorer response to neoCRT [7].

However, different ethnicities may have different allele frequencies for a SNP. It is not clear whether SNP rs1800925 in *IL13* has an association with response to neoCRT in Han Chinese patients. In this study, we explored the association between SNP rs1800925 (*IL13* 1112 C/T) and response to neoCRT and prognosis for 58 Chinese Han patients with LARC treated with neoCRT and surgery.

## RESULTS

### Pathological assessment of surgically resected specimen

Among the 58 patients included in this study, 28 (48.3%) were good responders:12 were TRG3 and 16 were TRG4 (15 patients had pCR, and 1 had pCR at the rectum primary tumor but had pN1a disease for postoperative lymph node staging). Thirty (51.7%) patients were poor responders: 8 displaying dominant tumor mass (i.e. TRG1) and 22 with few tumor cells or groups (TRG2) at rectal primary lesion.

### Correlation between rs1800925 polymorphisms and responsiveness to neoCRT

Of the 58 cases in this study, DNA from 8 formalin-fixed, paraffin-embedded specimens could not be successfully extracted, and a genotype could not be determined for 4 samples. The other 46 patients' DNA from paraffin-embedded specimens were successfully sequenced; 23 of them were good responders (11 patients with pCR) and the other 23 were poor responders. The genotypes of rs1800925 polymorphism were as follows: 34 cases with C/C genotype, 6 with T/T, and 6 with C/T. Table 2 shows the comparison between good responders and poor responders; no difference was observed between them. Table 3 shows the comparison of rs1800925 polymorphisms between patients with pCR and non-pCR; there was also no difference between these two groups (*P* > 0.05).

**Table 1:** Patient characteristics and their association with responsiveness to neoadjuvant chemoradiotherapy

Parameter	Cases (n)	Response to RT (n, %)		*P* value
		Good	Bad	
Gender				
Male	41	21 (51.2)	20 (48.8)	0.570
Female	17	7 (41.2)	10 (58.8)	
Tumor grade				
Well-moderatelydifferentiated	26	11 (42.3)	15 (57.7)	0.753
Poor differentiated	16	8 (50.0)	8 (50.0)	
Gx	16	9 (56.3)	7 (43.7)	
Pretreatment UICC TNM stage				
II	13	7 (53.8)	6 (46.2)	0.757
III	45	21 (46.7)	24 (53.3)	
Radiotherapy (Gy)				
46	52	27 (51.9)	25(48.1)	0.195
50	6	1 (16.7)	5 (83.3)	
Postoperative UICC TNM stage				
ypT				
ypT0-2	26	21 (80.8)	5 (19.2)	<0.001
ypT3-4	32	7 (21.9)	25 (78.1)	
ypN				
ypN0	38	20 (52.6)	18 (47.4)	0.416
ypN+	20	8 (40.0)	12 (60.0)	

Abbreviations: RT= radiotherapy; UICC= International Union Against Cancer; Gx= tumor differentiation is not clear.

**Table 2:** Analysis of genotypes associated with responsiveness to chemoradiotherapy in 58 patients

Genotype	Response to neoCRT (n, %)		*P* value
	good responders	poor responders	
C/C	17 (73.9)	17 (73.9)	1.0
T/T+C/T	6 (26.1)	6 (26.1)	
Sequencing failure	5	7	

Abbreviation: neoCRT= neoadjuvant chemoradiotherapy

**Table 3:** Analysis of genotypes associated with pCR in 58 patients

Genotype	Response to neoCRT (n,%)		*P* value
	pCR	non-pCR	
C/C	8 (72.7)	26 (74.3)	1.0
T/T+C/T	3 (27.3)	9 (25.7)	
Sequencing failure	4	8	

### Association between rs1800925 genotypes and ypT/ypN staging

Table 4 shows the correlation between rs1800925 genotypes and different ypT/ypN staging in the 46 patients. There were no differences between groups (*P* > 0.05).

**Table 4:** Correlation between rs1800925 genotypes and ypT/ypN staging in 46 patients

Genotype	ypT/ypN staging (n, %)			
	ypT		ypN	
	ypT0-2	ypT3-4	ypN0	ypN+
C/C	13 (38.2)	21 (61.8)	23 (67.6)	11 (29.4)
T/T+C/T	5 (41.7)	7 (58.3)	6 (50.0)	6 (50.0)
*P* value	1.0		0.314	

### Correlation between clinical characteristics and response to neoCRT

No difference was found between the pathologic response and classical clinicopathologic parameters such as age, gender, tumor grade, pre-therapeutic TNM stage, preoperative radiation dose, and ypN staging (*P*> 0.05). Only ypT staging was associated with the pathologic response to neoCRT. Patients with good response to neoCRT usually had lower ypT staging, as showed in Table 1.

### Association between rs1800925 genotypes and survival

As of July 8, 2015, the median follow-up time was 56.8 months (range, 4.4-111.6 months). Survival analyses were performed for the 46 cases that had been successfully sequenced for rs1800925. Seventeen of them had progressed diseases, 1 with liver metastases, 8 with lung metastases, 1 with concurrent liver and lung metastases, 2 with pelvic relapse, 1 with outside regional lymph nodes metastases, 1 with concurrent pelvic relapse and outside regional lymph nodes metastases, 2 with multiple bone metastases, and 1 with brain metastases. There were 12 deaths (11 dying of cancer itself, 1 dying of primary liver cancer). There were no differences between C/C and T/T+C/T genotype groups for the 6-year rate of PFS (67.6% vs. 50%, *P*= 0.274; Figure 2) or OS (76.5% vs. 66.7%, *P*= 0.441; Figure 3).

**Figure 1:**
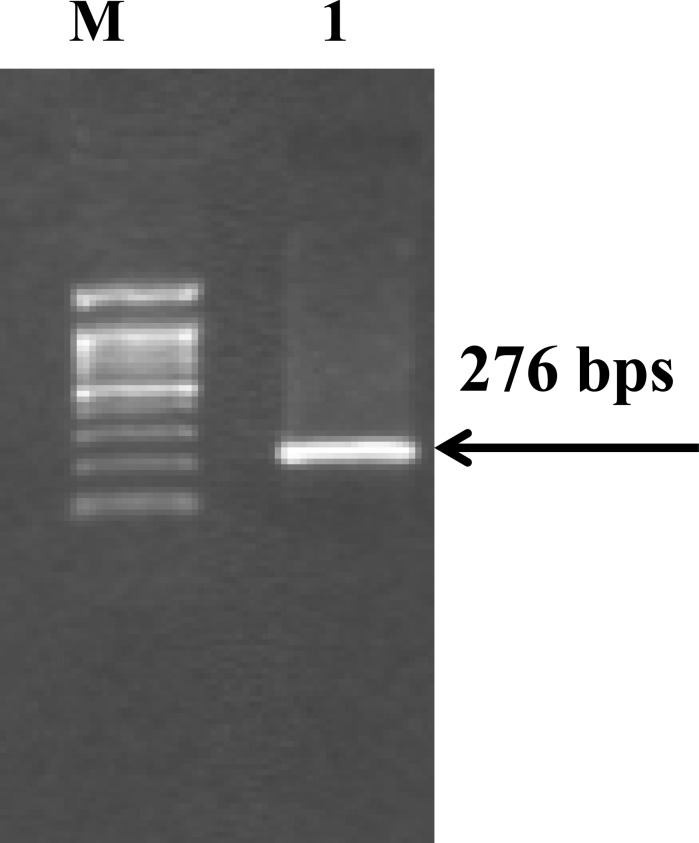
Amplified gene fragment of interest by PCR M= 100bp DNA Marker; Lane 1 = PCR products containing polymorphic site of rs 1800925

**Figure 2:**
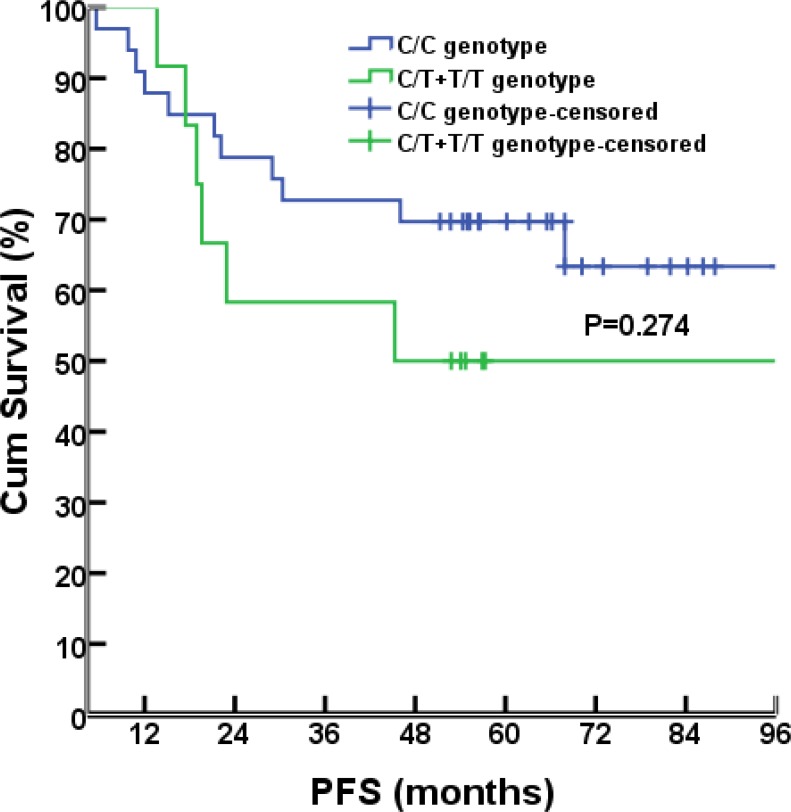
PFS comparison between different genotypes

**Figure 3:**
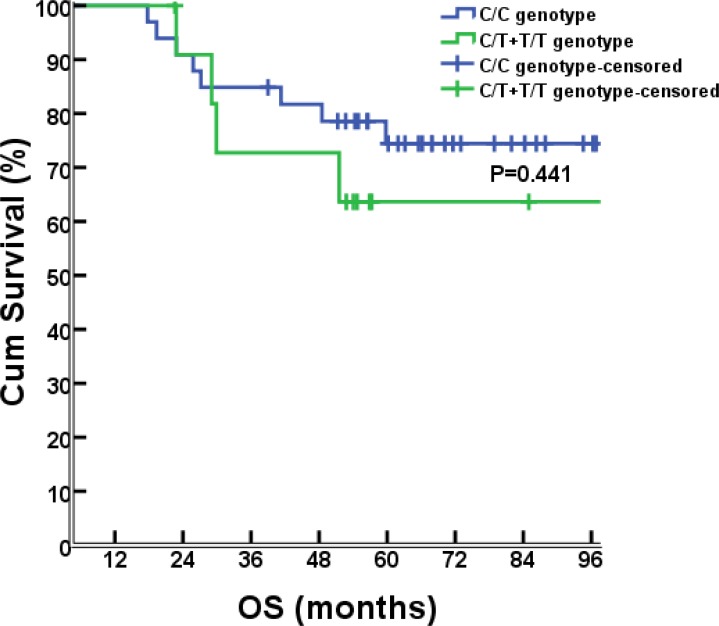
OS comparison between different genotypes

## DISCUSSION

Many studies have been focused on identifying predictive biomarkers of tumor response to neoCRT in patients with LARC [7,11,17–21, 30]. Yet some results are conflicting, even from studies focusing on the same biomarker. This may be partly attributed to different study methods and materials [31, 32]. Here, we successfully examined *IL13* rs 1800925 polymorphisms in 46 patients treated with neoCRT, and studied the association between rs 1800925 genotype and response to neoCRT, ypT/ypN staging, and prognosis. Our results were different from those reported by Ho-Pun-Cheung A et al [7].

*IL13* rs1800925 C/C genotypes from good responders and poor responders in our study equally accounted for 73.9% (17/23). However, in the results reported by Ho-Pun- Cheung A et al [7], *IL13* rs1800925 C/C genotype accounted for only 46.2% in the poor responders and 84.4% in the good responders. This difference may be attributed to the following factors: 1) Differences in ethnicity. In this study, all patients were Han Chinese from South China, while they were Caucasian in the previously published study. Some studies showed that Han Chinese from South China and North China had conflicting results for the same SNP genotype detection [33, 34]. 2) Differences of SNP genotyping methods. We used the gold-standard method of direct sequencing using an automatic sequencer, while in the study of Ho-Pun-Cheung A et al, the SNPlex Genotyping System (Applied Biosystems, Courtaboeuf, France) or the TaqMan allelic discrimination assay (Applied Biosystems) was used [7]. 3) Differences in genomic DNA extraction. In our study, the DNA was extracted from pre-therapeutic endoscopic biopsies of the primary rectum lesion, while it was from peripheral lymphocytes in the Ho-Pun-Cheung A et al's study [7].

The frequency of the T genotype in our study was 26.1%, which was lower than that reported by Ho-Pun-Cheung A et al [7] (36.6%). Some studies showed that the *IL13* rs1800925 C/C genotype was common, and that the T/T genotype was often seen in patients with asthma and allergic dermatitics [35, 36]. *IL13* rs1800925 T allele could increase IL-13 cytokine secretion, further stimulating the body's inflammatory response and immune suppression [23, 24, 37]. Ho-Pun-Cheung A et al [7] inferred that the rs1800925 T allele could inhibit chemoradiation-induced tumor immune surveillance and thereby decrease the chemoradiation effects by inducing higher IL13 transcription. However, this theory could not be supported by the results of our study.

Our study showed that different *IL13* rs1800925 genotypes (C/C vs. T/T+T/C) had no correlation with responsiveness to neoCRT, ypT/ypN staging or 6-year rate of PFS and OS for Han Chinese patients with LARC. Many clinicopathologic parameters such as age, sex, and tumor grade also had no significant association with postoperative pathologic response evaluation, and only ypT staging was associated with the pathologic response to neoCRT. These results are similar to those reported by Ho-Pun-Cheung A et al [7]. The degree of collinearity between ypT staging and responsiveness to neoCRT for patients with LARC may help to further understand their association.

A few limitations of this study need to be addressed. First, because it was a single-center retrospective study, selective bias cannot be completely avoided. However, we prospectively collected and systematically analyzed pre-therapeutic endoscopic biopsies of primary rectum lesions, so the bias may be limited. Second, the number of cases was too limited to help us to calculate a definitive minor allelic frequency of rs1800925 in the Chinese. Third, genomic DNA samples for genotyping in our study was from paraffin–embedded endoscopic biopsies of primary rectum lesions, rather than from peripheral lymphocytes. This meant that any somatic mutation or loss of heterozygoisty (LOH) in tumor cells themselves might potentially skew our final association analysis.

In conclusion, our study could not show a significant correlation between *IL13* 1112 C/T (rs 1800925) and responsiveness to neoCRT and prognosis of Chinese Han patients with LARC. Further studies may be focused on the combination of clinical parameters and prospectively screening genome-wide SNPs that are associated with the carcinogenesis and tumor progression.

## MATERIALS AND METHODS

### Ethics statement

This investigation was conducted in accordance with the ethical standards and according to the Declaration of Helsinki and according to national and international guidelines and was approved by the Sun Yat-sen University Cancer Center's Ethics Committee.

### Patients

Between January 2007 and September 2012, 58 Han Chinese patients with LARC who had histologically confirmed rectal adenocarcinoma and pre-treatment biopsy specimens in paraffin were retrospectively collected. There were 41 men and 17 women, with a median age of 58.5 years (range, 15-75 years) at the time of diagnosis. All patients underwent neoCRT. The 7th edition of the TNM staging standard of the American Joint Committee on Cancer (AJCC) was utilized to guide pre-treatment clinical staging and postoperative pathological staging [28]. All patients signed an informed consent form prior to treatment.

The pre-treatment clinical auxiliary examinations included blood counts, liver and renal function tests, CEA, endorectal ultrasonography (EURS), pelvic magnetic resonance imaging (MRI) and/or computed tomography (CT) scans, chest CT and/or X-ray; abdominal CT and/or MRI and/or ultrasound. A detailed description of the patients' clinicopathological characteristics and their response to chemoradiotherapy is presented in Table 1.

### Radiotherapy and concurrent chemotherapy

All patients were treated with megavoltage radiotherapy (6~8 MV) to the primary tumor and mesorectal, presacral, and internal iliac lymph nodes up to the level of the bottom of the fifth lumbar vertebra by a linear accelerator. Forty-two were treated using 3-dimensional conformal radiation therapy (3D-CRT) with one poster and two lateral fields, and received 46 Gy with a daily fraction of 2.0 Gy given 5 days per week. Six were treated using volumetric modulated arc therapy (VMAT), and received a concurrent boost to the tumor of 4 Gy with whole pelvic radiotherapy to a dose of 46 Gy. Ten were treated using 2-dimensional conventional radiotherapy with 3 fields of one poster and two lateral fields, and also received a total dose of 46 Gy with a daily fraction of 2.0 Gy given 5 days per week. All patients except 1 received concurrent chemotherapy during radiotherapy, 49 of them received 2 cycles of Xelox (capecitabine 1000 mg/m^2^ given twice daily on days1-14 plus a 2-hour intravenous infusion of oxaliplatin 100 mg/m^2^ on d1, every 3 weeks), 1 received 1 cycle of Xelox, 6 received 2 cycles of mFOLFOX6 (leucovorin plus oxaliplatin plus 5-FU).

### Surgery and surgical specimen evaluation

Surgery was performed at a median interval of 47 days after chemoradiotherapy (range, 29-76 days), observing principles of TME. The final choice of surgical procedure was at the discretion of the treating surgeon. The extent of the residual tumor in the resected specimen was evaluated according to the seventh International Union Against Cancer TNM staging system [28]. All patients were R0 resection, which was defined as histologically tumor-free.

### Response evaluation

Tumor response to neoCRT was evaluated by histopathological examination of the surgically resected specimen according to the tumor regression grade (TRG) system proposed by Dworak et al [29]. The resected specimen with primary caricinoma was embedded in paraffin and its corresponding TRG evaluation was semi-quantitatively determined by histopathologic examination of residual carcinoma cells vs. fibrosis or mucin pools. The TRG ranges from TRG 0 when no fibrosis is visible (no regression), to TRG 4 when no viable tumor cells are detected (i.e. complete regression). TRG 1 = dominant tumor mass and obvious fibrosis or mucin; TRG 2 = dominantly fibrotic or mucinous changes, with few tumor cells or groups; and TRG 3= very few tumor cells in fibrotic or mucinous tissue. Patients with TRG 0 to 2 were defined as poor responders, whereas those with TRG 3 to 4 were classified as good responders [21, 29].

### DNA extraction and SNP genotyping

For each patient, pre-therapeutic endoscopic biopsies of the primary rectum lesion were collected. Six to eight pieces of 4-mm-thick section were taken from formalin-fixed, paraffin-embedded blocks, and de-paraffinized; the remaining biologic materials were used for isolation of tumor DNA using a PureLink^TM^ Genomic DNA Mini Kit (Life Technologies^TM^ company) according to the manufacturer's recommendations. To determine the genotype at rs1800925, a fragment of 273 base pairs (bps) containing the polymorphic site of interest was amplified by 2 sequential polymerase chain reaction (PCR). The first PCR method was as follows: amplification was performed in a 25-μl volume containing 2.5μl of 10×PCR buffer (Invitrogen), 0.5μl of 10mM dNTP (Invitrogen), 0.8μl of 50mM MgCl_2_ (Invitrogen), 1 μl each of 5μM primer (forward: 5′-AGAGAGGGGGCCTGGG-3′) and (reverse: 5′-GTGATCCCCTTTGCTCACCA -3′), 1μl of biopsy-extracted DNA template, 0.2μl of Platinum^®^ Taq DNA polymerase (Invitrogen), and 18μl of ddH2O. The reaction was initiated by a pre-denaturation step at 94°C for 5 min, and then denaturation at 94°C for 30 sec, followed by 30 cycles at 94°C for 30 sec, 57°C for 30 sec, and 72°C for 30 sec, with a final extension at 72°C for 5 min. An additional PCR method was used to further amplify the initial PCR products. This reaction was also initiated by a denaturation step at 94°C for 5 min, followed by 40 cycles at 94°C for 30 sec, 57°C for 30 sec, and 72°C for 30 sec, with a final extension at 72°C for 5 min. The PCR products were visualized on a 2% agarose gel and stained with ethidium bromide (Figure 1) then digested by Shrimp Alkaline Phosphatase, purified, and directly sequenced using a DNA sequencer (ABI 3730XL, Applied Biosystems).

### Follow-up

All patients were evaluated regularly including clinical history, physical examination, laboratory investigations, EURS, pelvic MRI and/or CT scans, chest X-ray or CT, abdominal CT and/or MRI and/or ultrasound. They were followed postoperatively every 3 months in the first 2 years and then semiannually until death.

### Statistical analysis

Statistical analyses were performed using SPSS 16.0 statistical software (SPSS, Inc., Chicago, IL). Between-group proportional differences were compared using Fisher's exact test. Disease progression was defined as patients with pelvic primary lesion and/or regional lymph nodes relapse, and/or distant metastasis detected on imaging. Progression free survival (PFS) was defined as the time between the first of radiotherapy and the appearance of cancer progression. Overall survival (OS) was considered the interval between the first radiotherapy and last follow-up or death from any cause. Comparisons between the groups with regard to PFS and OS were performed using a log-rank test and Kaplan–Meier curves. A two-sided *P* value of ≤ 0.05 was considered statistically significant.
